# The Effects of 6 Isocaloric Meals Pattern on Blood Lipid Profile, Glucose, Hemoglobin A1c, Insulin and Malondialdehyde in Type 2 Diabetic Patients: A Randomized Clinical Trial

**Published:** 2014-09

**Authors:** Moosa Salehi, Asma Kazemi, Jafar Hasan Zadeh

**Affiliations:** 1 Department of Nutrition, School of Health and Nutrition, Shiraz University of Medical Sciences, Shiraz, Iran;; 2 Department of Epidemiology, School of Health and Nutrition, Shiraz University of Medical Sciences, Shiraz, Iran

**Keywords:** Diabetes mellitus, Blood glucose, Lipids

## Abstract

**Background: **The present clinical trial study aims at investigating the effect of daily energy intake in 6 isocaloric meals in comparison with the current meal pattern (3 meals and 2 small snacks per day) on type 2 diabetes risk markers in diabetes during 3-month period.

**Methods: **Eighty four type 2 diabetes patients were randomly divided into 6 isocaloric meal diet or a balanced diet (3 meals and 2 snacks previous meal pattern). The planned reduced calorie diets for both groups were identical except for the meal pattern. Blood samples were analyzed before and after the investigation for fasting blood sugar (FBS), two-hour post-prandial glucose (2hPP), insulin, hemoglobin A1c (HbA1c), total cholesterol, triglyceride, HDL-C, LDL-C, and molondialdehyde (MDA) concentrations.

**Results: **HbA1c (P=0.00) and body mass index (BMI) (P=0.04) values decreased significantly in the 6 isocaloric meal pattern compared with the controls. There were no significant differences in fasting serum glucose (P=0.09), insulin (P=0.65), total cholesterol (P=0.32), LDL-C (P=0.43), HDL-C (P=0.40) cholesterol, triglyceride (P=0.40), MDA (P=0.13) and 2hPP serum glucose (P=0.30) concentrations between the 6 isocaloric meal and tradition meal pattern.

**Conclusion: **Six isocaloric meal pattern in comparison with the current meal pattern led to weight loss and improved glycemic control. Serum lipid profile and MDA did not change significantly.

**Trial Registration Number: **IRCT201205179780N1

## Introduction

Type 2 diabetes is a consequence of resistance to the action of insulin resulting in hyperglycaemia. What is meant by macronutrients metabolism is varied.^1^ Nutrition therapy is a part of optimal treatment of diabetes.^2^ A dietary approach is modifying the frequency of meals; i.e. not only the amount and quality of food but also the number of feeding would affect metabolism.^1^ Several physiological reasons are suggested for the association between diabetes and meal frequency; spreading of nutrients throughout the day (which is seen in the pattern with more meals) induces lower glycaemic load.^3^ There is also a probable inverse association between glucose-dependent insulinotropic polypeptide and meal frequency.^4^ Higher small meal frequency leads to lower stomach extension and lower rate of stomach discharge, so the nutrients are delivered to the intestine with lower rates. As a consequence, less insulin is required to control the glucose.^4^ In lower meal frequency pattern there is a long interval between meals. In this state, the glycogen stores are depleted, so adipose tissue begins to lyse for meeting energy. As a consequence, the level of free fatty acids (FFA) in serum increases. High level of FFA in serum induces resistance to insulin and decrease in glucose tolerance. Also, after consuming a large meal, the level of FFA in the serum is high.^5^ Increasing meal frequency may improve serum lipid profile. Insulin has a stimulation effect on enzymes that are involved in lipogenesis and cholesterol synthesis. If insulin production decreases as a consequence of increasing meal frequency, serum lipid profile will be improved.^5^ The inverse association between increasing meal frequency and weight may be related to appetite control and increasing thermal effect of food (TEF)^6^ following an increase in meal frequency.

There are few studies that investigated the effect of meal frequency on diabetes risk markers. Results of these studies are inconsistent and inconclusive,^2^ since majority are of short duration and/or contain small sample size.^7^ Furthermore, these studies were mainly conducted on healthy individuals.^2^ This aspect of the proposed dietary habits (i.e. the effect of meal frequency) has not been investigated rigorously. Subsequently it is not possible to reach a conclusive result unless controlled studies are performed. The duration in most of the studies that investigated the effect of meals frequency on diabetic risk markers was short (e.g. five weeks or less). It is not clear if longer monitoring period of high frequency meal pattern will improve glucose tolerance.^2^ The current study aims at investigating the effect of 6 isocaloric meals pattern in comparison with the usual meal pattern of 3 meals and 2 small snacks per day on type 2 diabetes risk markers in diabetes for 3-month duration.

## Patients and Methods

*Ethical Approval*

This study was approved by Ethics Committee of Shiraz University of Medical Sciences and informed written consent was obtained from each subject.

*Study Design*

**Figure 1:**
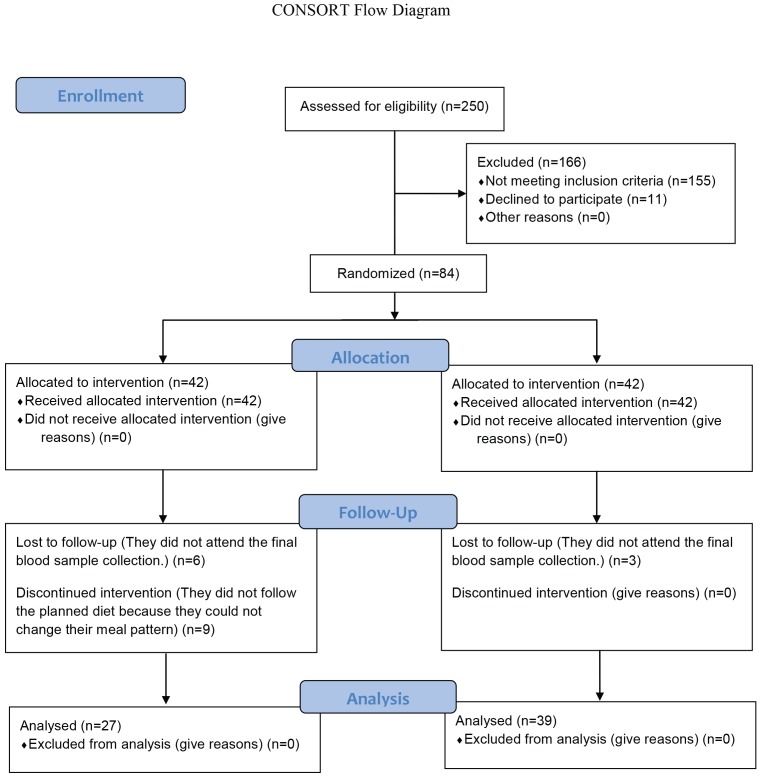
Flowchart of design and protocol of the study.

The study protocol and design is shown in figure 1. It is a randomized clinical trial with 3-moth follow up period in patients with type 2 diabetic. Eighty four volunteers were randomized into intervention or control groups. Randomization was performed in two stages, where initially stratified randomization was carried out based on age, gender and duration of diabetes. This was then followed by a simple randomization within the strata using a sequential list based on tables of random numbers.

*Population*

To calculate the sample size, the following formula was used. The sample size of 84 individuals was calculated based on the differences of fasting blood sugar (FBS) value observed in a study by Keshavarz et al.^8^ To take possible errors into account, the sample size was defined as 89 people.

$$n=\frac{2\delta^{2}{\left({\text{Z}}_{1-\frac{\alpha }{2}}+{\text{Z}}_{1-\beta }\right)}^{2}}{\left(\mu_{1}-\mu_{2}\right)}$$

$$\text{Power}\;\left({\text{Z}}_{1-\beta }\right)=\text{80\%,}\;\alpha =\text{\%5}$$

250 volunteers were enrolled to participate in the study. The exclusion criteria were; diabetes for more than 15 years, consumption of alcohol, vitamin supplements or hormonal medication, smoking, chronic or acute renal failure and finally liver or pulmonary diseases. Participants were required to have FBS≥126 mg/day and controlled blood glucose status. Among the total number of volunteers, 155 individuals with diabetes duration of less than 15 year were excluded. Among the 95 individuals who met the inclusion criteria, 11 declined participation and eventually a total of 84 candidates participated in this investigation. All subjects had stable weight with age ranging from 35 to 65 years. 

*Study Diets*

The planned diet for the intervention group was a balanced diet with 6 isocaloric meals per day (9-hour interval between the last meal and the next day’s first meal). The control group followed a balanced diet with their former number of meals (3 large meals with 2 small snacks). The calculated calorie for each subject in both groups was 300 Kilocalories (kcal) less than the required energy for weight maintenance. This was calculated based on gender, height, age and weight. Energy proportion from macronutrients were identical between the groups (approximately 56% for carbohydrate, 16% for protein and 28% for fat). The number of servings (sev) from each food group were identical for a definite calorie in both groups. For example a 1500 kcal diet for intervention group contained 7 sev bread, 2 sev milk, 4 sev fruit, 3 sev vegetable, 4 sev meat and 4 sev fat:

- 1st meal: 1 bread, 1 meat and substitutes, 0.5 milk, 1 fruit, 0.5 vegetable
- 2nd meal: 1 bread, 1 fat, 0.5 milk, 1 fruit
- 3rd meal: 1.5 bread, 1 meat and substitutes, 1 fat, 1 vegetable
- 4th meal: 1 bread, 1 meat and substitutes, 0.5 milk, 1 fruit
- 5th meal: 1.5 bread, 1 meat and substitutes, 1 fat, 1 vegetable
- 6th meal: 1 bread, 1 fat, 0.5 milk, 1 fruit, 0.5 vegetable

The planned diet for an individual with the same calorie (1500 kcal) in the control group contained 7 sev bread, 2 sev milk, 4 sev fruit, 3 sev vegetable, 4 sev meat and 4 sev fat, without being distributed into six meals. 

Eating behavior of participants was assessed using 24-h food recall. The intervention group was trained on how to distribute daily energy into 6 isocaloric meals. However, the control group only received training on healthy nutrition without mentioning the probable effect of meal frequency. The educational sessions were held separately for each group and they were called upon for blood sample collection on separate days. Each person was requested to record days in which diet was not followed. Both groups were contacted every two weeks to assess their compliance on the amount of food they consumed in each meals and the time of the meal on the previous day. The level of physical activity was nearly similar for both groups. Drug regimens for all individuals were recorded before and after the study. Their weighed was measured on an electronic scale and height with SECA meter before and after the 3-month duration of the study.

*Biological Sample Collection and Analysis*

Venous blood samples were taken using venous retention needles before and after the study. The samples were analyzed for FBS, two-hour post-prandial glucose (2hPP), insulin, hemoglobin A1c (HbA1c), total cholesterol, triglyceride, HDL-C, LDL-C and malondialdehyde (MDA) concentrations. 

The HbA1c measurement was made using ion exchange in EDTA anticoagulated whole blood (Bio-Rad DIAMAT). The serum glucose was measured with an enzymatic colorimetric test (ACCU-CHEK Active, Roche, Shanghai, China). Serum insulin levels were determined using the enzyme-linked immunosorbent assay technique (Boehringer Mannheim GmbH, Germany). Plasma total cholesterol, HDL-C, LDL-C and triglycerides were measured based on photometric method (BT 1500 AUTOANALYZER). Blood was assayed for malondialdehyde concentrations using a Beckman-Coulter DU 400 spectrophotometer (Fullerton, CA). To eliminate potential errors due to day-to-day laboratory variances, all blood samples were analyzed in a single batch following the completion of the study.

*Statistical Analysis*

Data were analyzed in SPSS statistics software, version 16. As all variables were normally distributed, paired *t* test was used for comparison of the measurements in each group before and after the study. For comparison of the measurements between groups, independent *t *test was used.

## Results

Forty two subjects participated in each group for this study. During the investigation, six individuals in the intervention group and three in the control group did not continue due to personal issues. Nine individuals in the intervention group did not follow the planned diet. Ultimately, 27 in the intervention and 39 in the control group completed the study. Five participants in the control group, and four in the intervention group were males and the rest were females. The Mean±SD values for the duration of diabetes were 4.83±2.64 for the intervention group and 5.24±3.04 for the control group. All participants had stable drug regimens. Drug regimens for both groups before intervention are presented in table 1. There were insignificant differences in parameters between both groups before the intervention.

**Table 1:** Drug regimen in both groups before the intervention

**Drug**	**Control**	**Intervention**	**P value**
Metformin	2.57±1.46	2.88±1.52	0.24
Gelibenclamide	1.31±1.47	1.66±1.94	0.49
Atrovastatin or other lipid lowering drugs	0.51±0.70	0.44±0.61	0.71

*BMI*

BMI significantly decreased on 6 isocaloric meals in comparison with the healthy balanced diet with usual meal pattern (3 large meals plus 2 small snacks) (P=0.04). Both meal patterns created significant changes in BMI values.

*Glycemic Control Markers*

HbA1c values decreased significantly in those on 6 isocaloric meals pattern compared with baseline and with those on usual meal pattern (P=0.00). The two meal patterns created insignificant changes in fasting serum glucose. The 6 isocaloric meal pattern significantly decreased insulin (P=0.01) and 2hpp (P=0.03) glucose concentrations. The usual meal pattern created insignificant changes in 2hPP (P=0.31) but decreased insulin concentration significantly (P=0.00). There were insignificant differences in fasting serum glucose (P=0.09) and insulin (P=0.48) and 2hPP serum glucose (P=0.30) concentrations between 6 isocaloric meals and the usual meal pattern. 

*Lipid Profile*

The usual meal pattern caused insignificant changes in serum TG as well as the total and LDL cholesterol values. Serum HDL concentration was significantly decreased after each of the two meals patterns. The 6 isocaloric meal pattern decreased the total and LDL cholesterol significantly and created insignificant change in TG value. There were insignificant differences in total cholesterol (P=0.32), LDL (P=0.43), HDL (P=0.40) and TG (P=0.40) concentrations between the two meal patterns.

*MDA*

Serum malondialdehyde concentrations did not change in response to the change in meal frequency (P=0.13). Meal patterns created insignificant changes in MDA values.

## Discussion

This study is among the first randomized control clinical trials investigating the effects of 6 isocaloric meals on diabetes risk markers. Previous studies have conducted mainly on healthy obese/normal weight individuals. In few studies that have investigated the effect of the number of meals on diabetes risk markers, study population was none diabetic individuals, meals were not isocaloric or the sample size was small. Furthermore, for the first time, this study investigates the effect of meals frequency on glycemic control in diabetes using HbA_1_c concentrations. The results indicate a decrease in HbA1c but not in other markers of glycaemic control (serum FBS, 2hpp glucose and insulin levels) following 6 isocaloric meals pattern for 3 months compared with the usual meal pattern. However, 6 isocaloric meals pattern decreased 2hPP and insulin serum significantly. In a review by American Dietetic Association in 2010, HbA1c is the clinical reported outcome since it is consistently mentioned in all studies.^2^ Considering the fact that other markers of glycemic control are not as rigorous as HbA1c, current study indicates that 6 isocaloric meal pattern induces improved glycaemic control in comparison with the usual meal pattern (3 non-isocaloric meals plus 2 small snacks). Larger sample size or weight maintenance interventions might be required to find improvement in FBS, 2hPP and insulin as a consequence of increasing meal frequency.

Twelve studies have investigated the effect of meal frequency on risk markers for diabetes. Among them, four studies were interventions for weight-loss.^9^^-^^12^ Young et al. found that glucose tolerance reduced on a weight-loss diet (one meal for a period of 5 weeks) indicating the adverse effect of diets with lower meal frequency.^12^ In other three weight-loss interventions, no relationship was found between serum glucose and insulin levels with meal frequency.^9^^-^^11^ All of the three studies were randomized control trials and compared 3-4 meals with 6 meals. Duration of the intervention in two of the randomized control trials was 24 months or more. Jenkin et al. in their weight maintenance intervention, indicated that mean insulin levels were 28% lower after 2 weeks on a 17-snack meal compared with the three meal diet.^13^ However, this study was limited as it was only carried out on seven cases. In two other weight maintenance studies, glucose/insulin curves were flatter in the intervention with more meals but the area under the curve was identical.^14^^,^^15^ In five other weight maintenance studies, with a duration of 5 weeks or less, no association was observed between meal frequency and diabetes risk markers.^16^^-^^20^

BMI was significantly decreased in 6 isocaloric meals in comparison with the current usual meal pattern. The results of studies investigating the effect of meal pattern on weight-loss are inconsistence.^6^^,^^2^ Findings of most previous studies are limited due to the short duration and small sample size.^8^

The effect of 6 isocaloric meals pattern on serum lipid profile values in comparison with the usual meal pattern were not significantly different. However, 6 isocaloric meals pattern significantly decreased the total and LDL cholesterol. Pervious interventions focusing on the effect of meal frequency on serum lipid profile were done on non-diabetic subjects.^3^ Most of the weight-loss studies that measured lipids, found no association between eating frequency and lipid levels. On the contrary, most of the weight-maintenance studies found an inverse association between blood lipids and eating frequency.^2^ It might be that the metabolic advantages of higher eating frequency blunted during standard weight-loss intervention as they already provide a reduced glycemic load. Thus, it is probable that weight-loss design of current intervention have blunted metabolic advantages of higher eating frequency. Jenkin et al.^10^ believes that large differences in eating frequency (8 or more) may be required to observe the association. Consequently, the difference in feeding frequency between the two groups in the current study might be too small to detect changes in blood lipid profile levels. However, our goal was to investigate a frequency that is realistic for daily living of humans. Unchanged TG level despite weight-loss may be due to the effect of weight change on TG level (and other metabolic syndrome) in Middle Eastern Caucasians being different from Western and Asian population.^21^ Serum TG level did not change significantly following both diets. Zabetian et al. investigated the effect of weight change on metabolic syndrome among the Iranian men and women in their cohort study. It was observed that weight-loss did not decrease TG level.^21^

The indicated decrease in the serum levels of malondialdehyde was not significantly different between the two groups. While there are no studies confirming the effect of meal frequency on oxidative stress markers, only a few have investigated post-prandial glucose consequent to meal size and at different intervals after a meal. It is indicated that larger meal size is accompanied with higher post-prandial glucose and higher serum malondialdehyde.^22^

## Conclusion

The 6 isocaloric meals pattern in comparison with the current meal pattern led to weight-loss and improved glycemic control. Serum lipid profile and MDA did not change significantly. 
